# Visual-Inertial Odometry with Robust Initialization and Online Scale Estimation

**DOI:** 10.3390/s18124287

**Published:** 2018-12-05

**Authors:** Euntae Hong, Jongwoo Lim

**Affiliations:** Division of Computer Science and Engineering, Hanyang University, Seoul 133-791, Korea; hongeuntae@hanyang.ac.kr

**Keywords:** visual-inertial odometry, UAV navigation, sensor fusion, optimization

## Abstract

Visual-inertial odometry (VIO) has recently received much attention for efficient and accurate ego-motion estimation of unmanned aerial vehicle systems (UAVs). Recent studies have shown that optimization-based algorithms achieve typically high accuracy when given enough amount of information, but occasionally suffer from divergence when solving highly non-linear problems. Further, their performance significantly depends on the accuracy of the initialization of inertial measurement unit (IMU) parameters. In this paper, we propose a novel VIO algorithm of estimating the motional state of UAVs with high accuracy. The main technical contributions are the fusion of visual information and pre-integrated inertial measurements in a joint optimization framework and the stable initialization of scale and gravity using relative pose constraints. To account for the ambiguity and uncertainty of VIO initialization, a local scale parameter is adopted in the online optimization. Quantitative comparisons with the state-of-the-art algorithms on the European Robotics Challenge (EuRoC) dataset verify the efficacy and accuracy of the proposed method.

## 1. Introduction

In robots and unmanned aerial vehicle systems (UAVs), the ego-motion estimation is essential. To estimate the current pose of a robot, various sensors such as GPS, inertial measurement units (IMU), wheel odometers, and cameras have been used. In recent years, the visual-inertial odometry (VIO) algorithm, which fuses the information from a camera and an IMU, has been garnering increasing interest because it overcomes the shortcomings of other sensors and can operate robustly. For example, a GPS sensor can estimate the global position of the device, but it can only operate in outdoors and cannot get precise positions needed for autonomous UAV navigation. An IMU sensor measures acceleration and angular velocity at high frequency, but the pose estimated by integrating the sensor readings easily drifts due to the sensor noise and time-varying biases. Visual odometry (VO) is more precise than other methods for estimating the device poses because it utilizes the long-term observations of fine visual features. However, it is vulnerable to motion blur from fast motions, the lack of scene textures, and abrupt illumination changes. Furthermore, monocular VO systems cannot estimate the absolute scale of motion due to the theoretical limitation of the camera’s projective nature. By fusing IMU and visual information, VIO operates in extreme environments where the VO fails and achieves higher accuracy with metric scale.

Initially, VIO was approached by loosely-coupled fusion of visual and inertial sensors [1,2]. An extended Kalman filter (EKF) [3,4] is also used, as it can update the current state (e.g., the 3D pose and covariance) by solving a linearized optimization problem for all state variables in a tightly-coupled manner [5,6,7]. The filtering-based approaches can estimate the current poses fast enough for real-time applications; however, they are less accurate than the optimization-based approach because of the approximation in the update step. Recently, optimization-based algorithms [5,6,7,8] have been developed for higher accuracy, but they require higher computational cost and suffer from divergence when the observation is poor or the initialization is not correct. Certainly, there is a trade-off between performance and speed, and it is difficult to optimize all the parameters in the initialization and update phase, especially when the information is insufficient.

In this work, we propose a VIO system that uses the tightly-coupled optimization framework of the visual and pre-integrated inertial observation, together with a robust initialization method for the scale and gravity. For real-time operation, the optimization cost for the trajectory estimation should not contain a large number of parameters. By using the pre-integrated IMU poses as the inertial costs, the number of pose parameters in the optimization window is drastically decreased, roughly from the number of frames to the number of keyframes. This reduction enables us to increase the size of the optimization window, which results in improved accuracy and robustness of the system. To account for the noise and error in the IMU biases, we introduce a local scale parameter in the device pose formulation.

Bootstrapping a VIO system requires careful treatment, as incorrect system parameters can easily break the system. The pose estimation problem for visual-inertial systems may not have a unique solution depending on the types of motion [9], and it makes the initialization task more challenging. As the IMU readings contain time-varying biases, we do not use the initial IMU measurement for the motion scale estimation. Instead of assuming that the biases are given to the system, we start with an arbitrarily-scaled vision-only map and upgrade it to a fully-metric map when enough information on the bias is available. We propose an efficient method to compute the global scale and gravity direction in the bootstrapping stage, by combining the relative pose constraints in the optimization. Furthermore, the convergence criterion to determine when to upgrade to the metric map and finish the bootstrapping process is proposed. As it works without any assumption on the motion or biases, this greatly improves the applicability of the proposed algorithm in the real world.

The experiments with the EuRoC [10] benchmark dataset confirm that our algorithm can estimate the reliable device poses with the correct real scale even in dynamic illumination changes and fast motions. On top of the robustness, we achieve better estimated pose accuracy compared to the state-of-the-art VIO algorithms. Our main contributions are summarized as follows:

- We propose a novel visual-inertial odometry algorithm using non-linear optimization of tightly-coupled visual and pre-integrated IMU observations with a local scale variable. The old information and estimation results are marginalized and utilized in the optimization for better stability.
- A robust online initialization algorithm for the metric scale and gravity directions is introduced. By enforcing the relative pose constraints between keyframes acquired from visual observations, the initial scale and gravity vectors can be estimated reliably, without assuming any bootstrapping motion patterns or that the bias parameters are given. To avoid failure due to the divergent scale variable in the optimization, we also propose a criterion that can determine the initialization window size adaptively and autonomously.
- The experimental results show that the proposed method achieves higher accuracy than the state-of-the-art VIO algorithms on the well-known EuRoC benchmark dataset.

## 2. Related Work

The VIO algorithms focus on highly accurate pose estimation of a device by fusing visual and IMU information. Cameras provide the global and stationary information of the world, but the visual features are heavily affected by the external disturbances like fast motion, lighting, etc. IMU sensors generate instantaneous and metric motion cues, but integrating the motions for a long period of time results in a noisy and drifting trajectory. As these two sensors are complementary, there have been many attempts to combine the two observations.

Recent VIO algorithms can be classified into the filtering-based approach, which feeds the visual and inertial measurements to filters, and the optimization-based approach, using non-linear optimization for state estimation. The former approaches use an extended Kalman filter (EKF) [11], which represents the state as a normal distribution with the mean and covariance. The EKF-based systems are faster than the optimization-based methods since they use linearized motion and observation models. In the multi-state constrained Kalman filter (MSCKF) [3], the visual information and IMU data are combined into a filter and the body poses are updated by a 3D keypoint processing with high accuracy. Li and Mourikis [4] proposed the new closed-form representation for the IMU error state transition matrix to improve the performance of MSCKF and the online model with extrinsic calibration. Hesch et al. [12] developed an observability constraint, OC-VINS, that explicitly enforces the system’s unobservable direction, to prevent spurious information gain and reduce discrepancies. The optimization-based methods are more accurate than the filtering-based method; however, they suffer from a high computational cost. To overcome this limitation, optimizing only a small window of poses or running an incremental smoothing is proposed [13,14]. Leutenegger et al. [5] proposed to calculate the position and velocity by integrating IMU measurements with VO’s keyframe interval while marginalizing out to old keyframe poses to mitigate complexity. However, these methods use the propagated poses of the IMU measurements for a certain interval, which has the disadvantage of re-integrating the linear acceleration value according to the device orientation changes for the local window. Forster et al. [8] proposed extending the IMU pre-integration method [15] to update the bias variables efficiently by calculating linear approximation IMU biases’ Jacobian for a very short interval using the IMU pre-integration method. Lupton and Sukkarieh [16] proposed a sliding window optimization framework for the IMU pre-integration method and old keyframe marginalization in the local window, and Qin and Shen [17] and Raul Mur-Artal and Tardos [6] combined VIO with the SLAM system for more accurate pose estimation.

The optimization methods directly use IMU sensor measurements together with the visual features as the constraints of the pose variables, which results in a highly non-linear formulation. For accurate and stable pose estimation, the initialization of the metric scale and gravity direction is critical because the time-varying IMU biases need to be calculated from the device poses. If the biases are not estimated accurately, the following online pose optimization is likely to diverge. Martinelli [9] demonstrated that there may exist multiple solutions in the visual-inertial structure from motion formulation. Mur-Artal and Tardos [6] proposed a closed-form formulation for vision-based structure from motion with scale and IMU biases; however, one should wait for initialization until 15 s to make sure all values are observable. Weiss et al. [18] proposed an initialization method that converges quickly using the extracted velocity and the dominant terrain plane based on the optical flow between two consecutive frames, but it requires aligning the initial pose and the gravity direction at the beginning. We discuss in Section 5 how to calculate the metric scale and gravity using the pose graph optimization (PGO) [19] and IMU pre-integration.

## 3. System Overview

**Figure 1:**
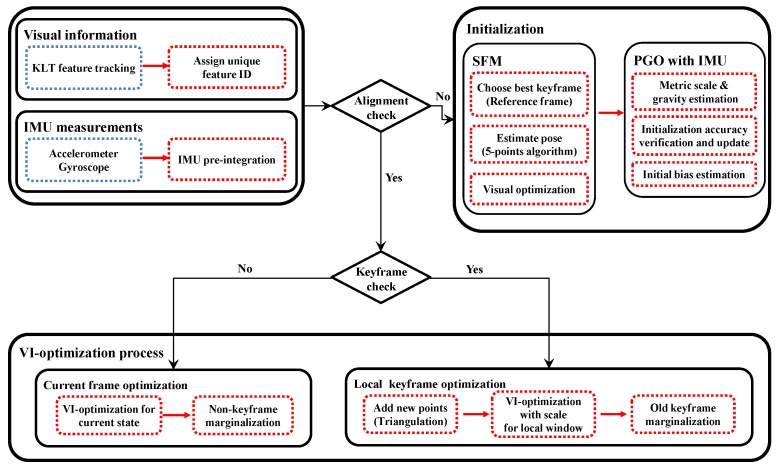
Overview of the proposed system. First, the initialization module computes a vision-only map and tries to determine the global metric scale and gravity. When this bootstrapping is over, the tightly-coupled VIO algorithm continuously estimates the device trajectory. PGO, pose graph optimization.

**Figure 2:**
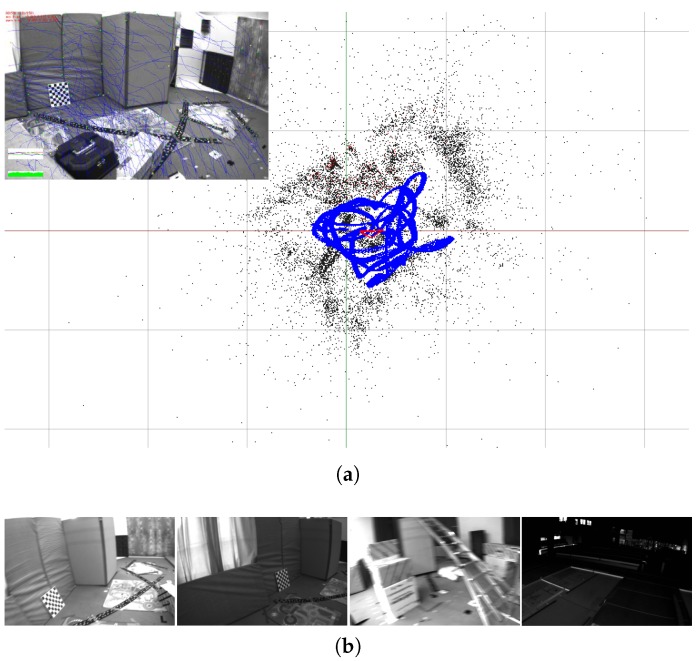
(**a**) An example result of the proposed system for V1-02 of the EuRoCbenchmark dataset. The blue line is the estimated trajectory; the black dots are the reconstructed sparse landmarks; and the red quadrangular pyramid represents the current camera pose. (**b**) Captured images in EuRoC with various challenges, such as motion blur and illumination changes. Our proposed system is able to estimate reliable poses for all sequences of EuRoC datasets (Section 6).

As shown in Figure 1, the proposed visual-inertial odometry algorithm consists of visual feature tracking, IMU pre-integration, initialization, and optimization modules. We use the Kanade–Lucas–Tomasi (KLT) feature tracker [20] to find the feature point correspondences for geometric modeling of camera poses and scene structure. Alternatively, one can use descriptor-matching algorithms [21,22,23,24] for this task, which also can be used for loop-closure finding in visual SLAM systems. We introduce a tightly-coupled visual-inertial odometry algorithm, which continuously estimates the motion state with a local scale parameter by minimizing the costs from visual information and IMU measurements (Section 4). For successful operation, it is critical to measure the IMU biases from the reliable metric poses and gravity direction. In Section 5, we present a robust initialization algorithm of the metric scale and gravity using pose graph optimization. Figure 2 shows one example result of our VIO system and a few images of the challenging situations from the EuRoC dataset. More results and discussions are presented in Section 6.

## 4. Visual Inertial Optimization

The goal of the visual-inertial odometer is to estimate the current motional state using visual information and inertial measurements at every time. The state $s_{t}$ at time *t* is defined as a quadruple:

(1) st=〈wdθt,wvt,dbat,dbωt〉,

where wdθ∈ special Euclidean group SE(3) is the rigid transformation parameter from the device to the world coordinate system, v is the velocity of the device, and ${}^{d}b^{a}$, ${}^{d}b^{\omega }$ are the sensor biases. The IMU sensor bias is modeled as a random walk, whose derivation is zero-mean and Gaussian as ${}^{d}{\dot{b}}^{a}=n^{b^{a}},{}^{d}{\dot{b}}^{\omega }=n^{b^{\omega }}$, where $n^{b^{a}}∼N\left(0,\sigma_{b_{a}}^{2}\right),n^{b^{\omega }}∼N\left(0,\sigma_{b_{\omega }}^{2}\right)$. The coordinate systems are denoted as a prescript on the left side of the symbol, and there are the world (w), the device (d), and the camera (c) coordinate systems. The time or keyframe index is denoted as a subscript (${}_{t}$ or ${}_{j}$) of the symbol. Let us denote the rigid transformation corresponding to θ as T=[R,p]∈SE(3), and ★ and ${}^{-1}$ denote the composition/application and the inversion operators for SE(3) transformations, respectively. The world coordinate system is defined so that the gravity direction is aligned with the negative *z*-axis. We follow the convention that the device coordinate system is aligned with the IMU coordinate system. The transformation from the camera to the device coordinate system is written as ${}_{c}^{d}T$, and it is pre-calculated in the device calibration process [25,26].

### 4.1. Visual Reprojection Error

**Figure 3:**
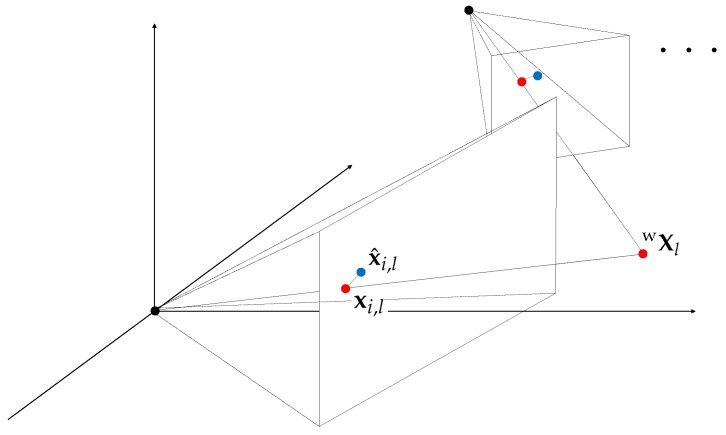
Illustration of the visual error. The green dashed line represents re-projection error $e^{\nu }$, and the visual error term optimizes the summation of these errors for the local window.

The visual error term of our proposed method uses the re-projection error in the conventional local bundle adjustment. The error is the difference between the projected location $x_{i,l}$ of a 3D landmark $X_{l}$ and its tracked location ${\hat{x}}_{i,l}$ at the keyframe *i*. As illustrated in Figure 3, the visual cost $C_{i,l}^{\nu }$ from the tracked features is defined as:

(2) $$C_{i,l}^{\nu }=\rho \left(e^{\nu }{\left(i,l\right)}^{⊤}\Lambda_{i,l}^{\nu }e^{\nu }\left(i,l\right)\right)$$

(3) $$e^{\nu }\left(i,l\right)={\hat{x}}_{i,l}-\pi \;\left({}_{d}^{c}T^{-1}⋆T{\left({}_{w}^{d}\theta_{i}\right)}^{-1}⋆{}^{w}X_{l}\right),$$

where $\Lambda_{i,l}^{\nu }$ is the information matrix associated with the tracked feature point at the keyframe and π denotes the camera projection function. ρ is the Huber norm [27], which is defined as:

(4) $$\rho \left(x\right)=\left\{\begin{array}{cc} 1, & \mathrm{if}\;x\geq 1\\ 2\sqrt{x}-1, & \mathrm{if}\;x<1 \end{array}\right..$$

### 4.2. IMU Pre-Integration

The IMU sensors measure the angular velocity and translational acceleration, and in theory, the 3D pose (orientation and position) of the device can be calculated by integrating the sensor readings over time. However, the raw IMU measurements contain significant noise and time-varying non-zero bias, and these make the integration-based pose estimation very challenging. The IMU angular velocity ${}^{d}\hat{\omega }$ and acceleration ${}^{d}\hat{a}$ measurements at time *t* are modeled with the true acceleration ${}^{w}a$ and angular velocity ${}^{d}\omega$ as:

(5) $${}^{d}{\hat{a}}_{t}={}_{d}^{w}R_{t}^{⊤}\left({}^{w}a_{t}-{}^{w}g\right)+{}^{d}{b^{a}}_{t}+n^{a},\;\mathrm{and}$$

(6) $${}^{d}{\hat{\omega }}_{t}={}^{d}\omega_{t}+{}^{d}{b^{\omega }}_{t}+n^{\omega },$$

where ${}_{d}^{w}R_{t}^{⊤}$ is the rotation from the world to the device coordinates (note the transpose), ${}^{w}g$ is the constant gravity vector in the world, ${}^{d}{b^{a}}_{t}$, ${}^{d}{b^{\omega }}_{t}$ are the acceleration and gyroscope biases, and $n^{a}$, $n^{\omega }$ are the additive zero-mean noise. From the following relations,

(7) $$\left[\begin{array}{c} {}^{w}\dot{p}={}^{w}v\\ {}^{w}\dot{v}={}^{w}a\\ {}^{w}\dot{R}={}_{d}^{w}R{\left[{}^{d}\omega \right]}_{\times } \end{array}\right],\;\;\mathrm{where}\;\;{\left[\omega \right]}_{\times }=\left[\begin{array}{ccc} 0 & -\omega_{z} & \omega_{y}\\ \omega_{z} & 0 & -\omega_{x}\\ -\omega_{y} & \omega_{x} & 0 \end{array}\right],$$

for the image frames *k* and k+1 (at time $t_{k}$ and $t_{k+1}$, respectively), the position, velocity, and orientation of the device can be propagated through the first and second integration used in [28],

(8) $${}^{w}p_{k+1}={}^{w}p_{k}+{}^{w}v_{k}\Delta t_{k}+\int \int_{t\in t_{k},t_{k+1}}\left({}_{d}^{w}R_{t}\left({}^{d}{\hat{a}}_{t}-{}^{d}{b^{a}}_{t}-n^{a}\right)+{}^{w}g\right)dt^{2}$$

(9) $${}^{w}v_{k+1}={}^{w}v_{k}+\int_{t\in t_{k},t_{k+1}}\left({}_{d}^{w}R_{t}\left({}^{d}{\hat{a}}_{t}-{}^{d}{b^{a}}_{t}-n^{a}\right)+{}^{w}g\right)dt$$

(10) $${}_{d}^{w}R_{k+1}={}_{d}^{w}R_{k}\;\mathrm{Exp}\left(\int_{t\in t_{k},t_{k+1}}\left({}^{d}{\hat{\omega }}_{t}-{}^{d}{b^{\omega }}_{t}-n^{\omega }\right)dt\right).$$

Assuming the acceleration ${}^{d}{\hat{a}}_{k}$ and the angular velocity ${}^{d}{\hat{\omega }}_{k}$ are constant between time interval $t_{k}$ and $t_{k+1}$, we can simplify the above equations as follows:

(11) $${}^{w}p_{k+1}={}^{w}p_{k}+{}^{w}v_{k}\Delta t_{k,k+1}+\frac{1}{2}{}^{w}g\Delta t_{k,k+1}^{2}+\frac{1}{2}{}_{d}^{w}R_{t_{k}}\left({}^{d}{\hat{a}}_{t_{k}}-{}^{d}{b^{a}}_{t_{k}}-n^{a}\right)\Delta t_{k,k+1}^{2}$$

(12) $${}^{w}v_{k+1}={}^{w}v_{k}+{}^{w}g\Delta t_{k,k+1}+{}_{d}^{w}R_{t_{k}}\left({}^{d}{\hat{a}}_{t_{k}}-{}^{d}{b^{a}}_{t_{k}}-n^{a}\right)\Delta t_{k,k+1}$$

(13) $${}_{d}^{w}R_{k+1}={}_{d}^{w}R_{t_{k}}\;\mathrm{Exp}\left(\left({}^{d}{\hat{\omega }}_{t_{k}}-{}^{d}{b^{\omega }}_{t_{k}}-n^{\omega }\right)\Delta t_{k,k+1}\right).$$

**Figure 4:**
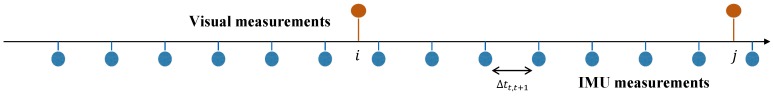
IMU sensor measurements are typically much faster than the camera frame rate. The EuRoC benchmark provides the IMU sensor readings at 200 Hz and camera images at 20 fps. *i* and *j* denote the time of camera capture, and *t* is the IMU measurement time.

The measurement rate of the IMU is much faster than that of the camera, as illustrated in Figure 4, and it is computationally burdensome to re-integrate the values according to the changes of the state in the optimization framework. Thus, we adopt the pre-integration method, which represents IMU measurements in terms of the poses of the consecutive frames by adding IMU factors incrementally as in [7,29].

For two consecutive keyframes [i,j] where the time between two ($t_{i},t_{j}$) can vary, the changes of position, velocity, and orientation that are not dependent to the biases can be written as follows from Equations (11)–(13):

(14) $$\Delta p_{i,j}:={}_{d}^{w}R_{i}^{⊤}\left({}^{w}p_{j}-{}^{w}p_{i}-{}^{w}v_{i}\Delta t_{i,j}-\frac{1}{2}{}^{w}g\Delta t_{i,j}^{2}\right)\;=\;\sum_{k=i}^{j-1}\frac{1}{2}R_{k}^{i}\left({}^{d}{\hat{a}}_{t_{k}}-{}^{d}{b^{a}}_{t_{k}}-n^{a}\right)\Delta t_{k,k+1}^{2}$$

(15) $$\Delta v_{i,j}:={}_{d}^{w}R_{i}^{⊤}\left({}^{w}v_{j}-{}^{w}v_{i}-{}^{w}g\Delta t_{i,j}\right)\;=\;\sum_{k=i}^{j-1}R_{k}^{i}\left({}^{d}{\hat{a}}_{t_{k}}-{}^{d}{b^{a}}_{t_{k}}-n^{a}\right)\Delta t_{k,k+1}$$

(16) $$\Delta R_{i,j}:={\left({}_{d}^{w}R_{i}\right)}^{⊤}{}_{d}^{w}R_{j}\;=\;\prod_{k=i}^{j-1}\mathrm{Exp}\left(\left({}^{d}{\hat{\omega }}_{t_{k}}-{}^{d}{b^{\omega }}_{t_{k}}-n^{\omega }\right)\Delta t_{k,k+1}\right),$$

where $R_{k}^{i}$ represents the rotation from the frame *k* to the time *i*. We can calculate the right side of above equation directly from the IMU measurements and the biases between the two keyframes. However, these equations are functions of the biases, ${}^{d}{b^{a}}_{t_{k}}$ and ${}^{d}{b^{\omega }}_{t_{k}}$. If the biases ${}^{d}b^{a}$ and ${}^{d}b^{\omega }$ between the keyframes are assumed to be fixed, we can obtain the values of $\Delta p_{i,j},\Delta v_{i,j},\Delta R_{i,j}$ from the IMU measurements without re-integration.

However, in the case of bias, it changes slightly in the optimization window, and we use the recent IMU pre-integration described in [7,29] to reflect the bias changes in the optimization by updating delta measurements of bias using the Jacobians, which describe how the measurements change due to the estimation of the bias. The bias is updated from the delta measurements $\delta b^{a}$ and $\delta b^{\omega }$ using the first-order approximation as,

(17) $$\Delta p_{i,j}≃\Delta {\bar{p}}_{i,j}+J_{\Delta p}^{\omega }{}^{d}{\delta b^{\omega }}_{i}+J_{\Delta p}^{a}{}^{d}{\delta b^{a}}_{i}$$

(18) $$\Delta v_{i,j}≃\Delta {\bar{v}}_{i,j}+J_{\Delta v}^{\omega }{}^{d}{\delta b^{\omega }}_{i}+J_{\Delta v}^{a}{}^{d}{\delta b^{a}}_{i}$$

(19) $$\Delta R_{i,j}≃\Delta {\bar{R}}_{i,j}\;\mathrm{Exp}\left(J_{\Delta R}^{\omega }{}^{d}{\delta b^{\omega }}_{i}\right),$$

where $\Delta {\bar{p}}_{i,j},\Delta {\bar{v}}_{i,j},\Delta {\bar{R}}_{i,j}$ are the pre-integrated measurements from the fixed bias and Jacobians $\left[J_{\Delta p}^{\omega },J_{\Delta v}^{\omega },\ldots \right]$ are computed at integration time, describing how the measurements change from bias estimation [29].

(20) $${}^{w}p_{j}={}^{w}p_{i}+{}^{w}v_{i}\Delta t_{i,j}+\frac{1}{2}{}^{w}g\Delta t_{i,j}^{2}+{}_{d}^{w}R_{i}\left(\Delta {\bar{p}}_{i,j}+J_{\Delta p}^{\omega }{}^{d}{\delta b^{\omega }}_{i}+J_{\Delta p}^{a}{}^{d}{\delta b^{a}}_{i}\right)$$

(21) $${}^{w}v_{j}={}^{w}v_{i}+{}^{w}g\Delta t_{i,j}+{}_{d}^{w}R_{i}\left(\Delta {\bar{v}}_{i,j}+J_{\Delta v}^{\omega }{}^{d}{\delta b^{\omega }}_{i}+J_{\Delta v}^{a}{}^{d}{\delta b^{a}}_{i}\right)$$

(22) $${}_{d}^{w}R_{j}={}_{d}^{w}R_{i}\;\Delta {\bar{R}}_{i,j}\;\mathrm{Exp}\left(J_{\Delta R}^{\omega }{}^{d}{\delta b^{\omega }}_{i}\right),$$

Finally, the local optimization cost of the IMU residual $e_{i,j}^{\mu }$ for the interval of keyframes *i* and *j* using pre-integration is defined as follows:

(23) $$C_{i,j}^{\mu }=e^{\mu }{\left(i,j\right)}^{⊤}\;\Lambda_{i,j}^{\mu }\;e^{\mu }\left(i,j\right)$$

(24) $$e^{\mu }\left(i,j\right)=\left[\begin{array}{c} {}_{d}^{w}R_{i}^{⊤}\left({}^{w}p_{j}-{}^{w}p_{i}-{}^{w}v_{i}\Delta t_{i,j}-\frac{1}{2}{}^{w}g\Delta t_{i,j}^{2}\right)-\left(\Delta {\bar{p}}_{i,j}+J_{\Delta p}^{\omega }{}^{d}{\delta b^{\omega }}_{i}+J_{\Delta p}^{a}{}^{d}{\delta b^{a}}_{i}\right)\\ {}_{d}^{w}R_{i}^{⊤}\left({}^{w}v_{j}-{}^{w}v_{i}-{}^{w}g\Delta t_{i,j}\right)-\left(\Delta {\bar{v}}_{i,j}+J_{\Delta v}^{\omega }{}^{d}{\delta b^{\omega }}_{i}+J_{\Delta v}^{a}{}^{d}{\delta b^{a}}_{i}\right)\\ \mathrm{Log}({\left(\Delta {\bar{R}}_{i,j}\;\mathrm{Exp}\left(J_{\Delta R}^{\omega }{}^{d}{\delta b^{\omega }}_{i}\right)\right)}^{⊤}{\left({}_{d}^{w}R_{i}\right)}^{⊤}{}_{d}^{w}R_{j})\\ {}^{d}{b^{a}}_{j}-{}^{d}{b^{a}}_{i}\\ {}^{d}{b^{\omega }}_{j}-{}^{d}{b^{\omega }}_{i} \end{array}\right]$$

where $\Lambda_{i,j}^{\mu }$ is the information matrix associated with the IMU pre-integration covariance between the keyframes, reflecting the IMU factor noise. The computed measurement of IMU pre-integration factor is a function of the random noises $\left[n^{a},n^{\omega },n^{b^{a}},n^{b^{\omega }}\right]$, which are assumed to be zero-mean and Gaussian. A covariance matrix of pre-integrated parameters $\Sigma_{i,j}^{\mu }\in R^{15\times 15}$ is propagated from the knowledge of the IMU sensor noise given in the sensor specifications. As the IMU biases follow the Brownian motion model, we penalize abrupt changes of the biases between consecutive keyframes with the bias costs at the bottom two entries in Equation (25).

### 4.3. Online Optimization

Considering UAVs, the VIO system should estimate the current pose in real time using captured visual information and IMU measurement. We use the visual-inertial bundle adjustment framework and solve the optimization problem with the Gauss–Newton algorithm implemented in Ceres Solver [30]. For the states $s_{k}$ and the 3D landmarks $X_{l}$, the cost function is defined as follows for the optimization window:

(25) $$S_{online}^{*}=\underset{\left\{s_{k}\right\},\left\{l_{i}\right\}}{\mathrm{argmin}}\left\{C^{\rho }+\sum_{\left(i,l\right)}C_{k,i}^{\nu }+\sum_{k=0}^{n-1}C_{k,k+1}^{\mu }\right\},$$

where $C^{\rho }$ is the prior information from marginalization, which is the factor for the states out of the local optimization window.

In order to estimate the best metric scale, we add the local scale factor $\;e^{s^{\prime }}$ into our cost function (Equation (26)) and optimize it together with other variables. When a new keyframe is added, we assume that the device experiences the motion changes and perform joint optimization including the local scale $s^{\prime }$ variable. To prevent the scale from becoming zero or negative, we use the exponential parameterization $e^{s^{\prime }}$ instead of using $s^{\prime }$ directly. The updated IMU residual is:

(26) $$e^{\mu }\left(i,j\right)=\left[\begin{array}{c} {}_{d}^{w}R_{i}^{⊤}\left(\;e^{s^{\prime }}\;\left({}^{w}p_{j}-{}^{w}p_{i}\right)-{}^{w}v_{i}\Delta t_{i,j}-\frac{1}{2}{}^{w}g\Delta t_{i,j}^{2}\right)-\left(\Delta {\bar{p}}_{i,j}+J_{\Delta p}^{\omega }{}^{d}{\delta b^{\omega }}_{i}+J_{\Delta p}^{a}{}^{d}{\delta b^{a}}_{i}\right)\\ {}_{d}^{w}R_{i}^{⊤}\left({}^{w}v_{j}-{}^{w}v_{i}-{}^{w}g\Delta t_{i,j}\right)-\left(\Delta {\bar{v}}_{i,j}+J_{\Delta v}^{\omega }{}^{d}{\delta b^{\omega }}_{i}+J_{\Delta v}^{a}{}^{d}{\delta b^{a}}_{i}\right)\\ \mathrm{Log}({\left(\Delta {\bar{R}}_{i,j}\mathrm{Exp}\left(J_{\Delta R}^{\omega }{}^{d}{\delta b^{\omega }}_{i}\right)\right)}^{⊤}{\left({}_{d}^{w}R_{i}\right)}^{⊤}{}_{d}^{w}R_{j})\\ {}^{d}{b^{a}}_{j}-{}^{d}{b^{a}}_{i}\\ {}^{d}{b^{\omega }}_{j}-{}^{d}{b^{\omega }}_{i} \end{array}\right].$$

**Figure 5:**
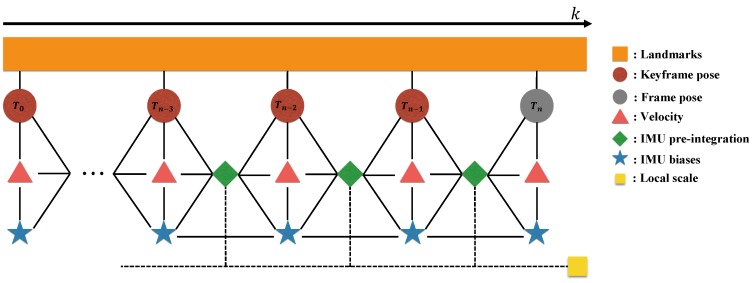
Illustration of the proposed visual inertial local bundle adjustment. All keyframe poses $\langle {}_{d}^{w}\theta_{0},{}_{d}^{w}\theta_{1},\ldots ,{}_{d}^{w}\theta_{n}\rangle$ contain the visual terms with landmarks and the IMU pre-integration factors with a common local scale parameter. The current frame *n* (which may not be a keyframe) is included in the local window with the accumulated IMU pre-integration.

**Figure 6:**
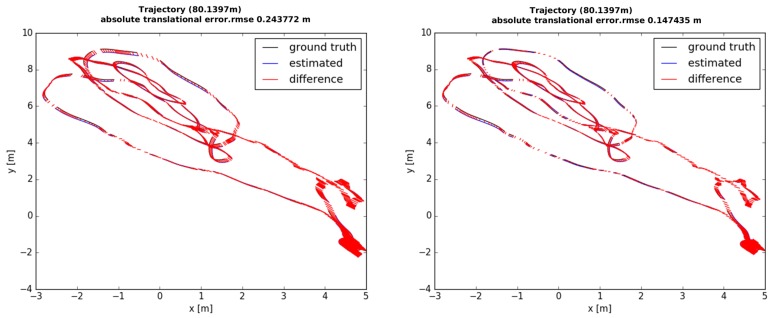
The difference in the trajectories from the ground-truth to the optimization without the scale (**left**) and the proposed optimization with the scale parameter (**right**). The estimated trajectories are aligned to the ground-truth via a rigid transform (special Euclidean group SE3) using the Technical University of Munich (TUM) RGB-D benchmark tool [31]. The proposed method is able to accurately estimate the poses by updating the scale incrementally.

Figure 5 shows the graphical model of our visual inertial local bundle adjustment. We perform local optimization with the sufficiently accurate scale variable computed by bootstrapping in Section 5, and the optimized local scale is marginalized to prior information along with the poses of the old keyframes. Figure 6 shows the comparison results with or without the local scale variable. Optimization involving local scale factor achieves accurate estimation of poses, since this approach is able to refine local scale information.

### 4.4. Marginalization

**Figure 7:**
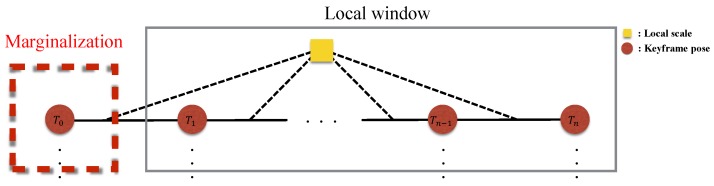
Marginalization of the old keyframes with local scale. Marginalized measurements are used as the prior for the next optimization.

The optimization-based VIO algorithms need to marginalize out the old information so as not to slow down the processing speed [5,7]. The marginalization does not eliminate the old information outside of the local optimization window of keyframes, but converts it into a linearized approximate form to the remaining state variables using the Schur complement [32]. When a new keyframe is added into the local optimization window and the window size exceeds the preset threshold, the state (the pose, velocity, and bias) of the oldest keyframe in the window is marginalized (Figure 7 illustrates keyframe marginalization in a graphical model). On the other hand, if the current frame is not selected as a keyframe, only the visual information is dropped, while the IMU measurements are kept for IMU pre-integration. The marginalized factor is applied to be a prior of the next optimization, which helps to find a better solution than simply fixing the keyframe poses outside of the optimization window.

## 5. Bootstrapping

Unlike the monocular visual odometry where the absolute scale of the map is not recoverable, the visual-inertial odometry needs to find the important parameters such as the scale of the map and gravity direction to estimate the metric state robustly. Moreover, there are many motion patterns in which the multiple solutions of IMU bias parameters exist, such as constant velocity motions including no motion [9]; thus, optimization involving all state variables without precise initialization may not converge to the true solution. For these reasons, some VIO systems require approximate manual initialization of the gravity vectors or IMU biases, or real scale distance information using different sensors [33]. The map of visual features is constructed starting from the two keyframes with sufficient parallax, and it is continuously updated as more keyframes are observed. However, the IMU measurements for these keyframes may not observe any significant changes in acceleration, and this can cause failure in bootstrapping the VIO system.

In this work, we propose a bootstrapping method that computes the accurate scale and gravity through stepwise optimization using relative pose constraints. Our method consists of vision-only map building, pose graph optimization with IMU pre-integration, convergence check, and IMU bias update.

### 5.1. Vision-Only Map Building

The first step, vision-only map building, is identical to monocular visual odometry [34,35] and structure from motion algorithms (SFM) [36]. The system finds the first two keyframes ${}_{c}^{w}T_{0}$ and ${}_{c}^{w}T_{1}$ with sufficient motion, by checking the numbers of inlier features by a homography and a fundamental by the five-point algorithm [37], as only the fundamental matrix can explain the non-planar scene with enough parallax depth, and it is important for reliable 3D point reconstruction [35]. If the absolute scale of motion is not available, the visual map is initialized with an arbitrary scale, and the inlier features are triangulated and their 3D positions registered. The gravity direction is roughly initialized with the average of the initial acceleration readings (we experimentally use the first 30 readings @200 Hz), and the world coordinate system is set by aligning the gravity to *y*-down. Once the initial map with 3D points is built, the poses of later keyframes are computed by the Perspective-n-Point (PNP) algorithm [38] Local bundle adjustment using Equation (2) is performed initially and whenever a keyframe is added to improve the accuracy of pose and point positions. Until the scale and gravity are reliably measured in the next steps, purely vision-only map building is continued.

### 5.2. Pose Graph Optimization with IMU Pre-Integration

**Figure 8:**
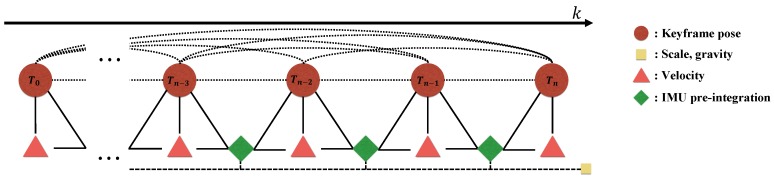
The proposed pose graph optimization model for bootstrapping. We estimate the global metric scale and gravity vector while maintaining the relative poses between keyframes computed only from the visual information.

While the purely-visual mapping is running, we try to estimate the metric scale using the pre-integrated IMU factor. For easy formulation and efficient estimation, we adopt the pose graph optimization (PGO) framework [19,39,40], which constructs a graph of keyframes where the edges represent the relative pose constraints between keyframes, and optimizes the keyframe poses so that the inconsistency of the relative poses and constraints are minimized (note that this is equivalent to marginalizing the landmarks in a standard bundle adjustment). PGO is commonly used in monocular SLAM systems to fix the scale drift in loop closures using Sim(3) relative poses. In contrast, we use SE(3) relative poses with a global scale parameter *s* for the entire map, as the scale drift for a short period of initialization time is not significant. Additional constraints from the pre-integrated IMU and the gravity vector are added to PGO, and the factors in our formulation are illustrated in Figure 8. Furthermore, to expedite the convergence, the gravity vector g is also included in the active parameters. Because the magnitude of gravity g is always 9.8, we include the constraint $g^{⊤}g=9.8^{2}$ when performing the optimization.

Formally, we define the state for PGO with all keyframe poses, velocities, the gravity, and the global scale *s* as:

(27) $$S_{pgo}=\langle {}_{d}^{w}\theta_{0},{}_{d}^{w}\theta_{1},\ldots ,{}_{d}^{w}\theta_{n},{}^{w}v_{0},\ldots ,{}^{w}v_{n},{}^{w}g,s\rangle .$$

In this section, we parameterize ${}_{d}^{w}\theta_{k}$ an SE(3) transformation with a pair of a translation vector p and a Hamiltonian quaternion [41] q, i.e., θ=[R(q),p], where R(·) is the function converting a quaternion to a 3×3 rotation matrix.

While performing visual pose estimation, we calculate IMU pre-integration for keyframes using Equations (20)–(22), in which bias and noise are initialized as zero. Using Equations (20) and (21) for consecutive keyframes *i* and *j*, we obtain the scale error cost $e_{i,j}^{s}$:

(28) $$C_{i,j}^{s}={\left(e^{s}\left(i,j\right)\right)}^{⊤}\Lambda_{i,j}^{s}e^{s}\left(i,j\right)$$

(29) $$e^{s}\left(i,j\right)=\left[\begin{array}{c} R{\left({}^{w}q_{i}\right)}^{⊤}\left(\;e^{s}\;\left({}^{w}p_{j}-{}^{w}p_{i}\right)-{}^{w}v_{i}\Delta t_{i,j}-\frac{1}{2}{}^{w}g\Delta t_{i,j}^{2}\right)-\left(\Delta {\bar{p}}_{i,j}+J_{\Delta p}^{\omega }{}^{d}{\delta b^{\omega }}_{i}+J_{\Delta p}^{a}{}^{d}{\delta b^{a}}_{i}\right)\\ R{\left({}^{w}q_{i}\right)}^{⊤}\left({}^{w}v_{j}-{}^{w}v_{i}-{}^{w}g\Delta t_{i,j}\right)-\left(\Delta {\bar{v}}_{i,j}+J_{\Delta v}^{\omega }{}^{d}{\delta b^{\omega }}_{i}+J_{\Delta v}^{a}{}^{d}{\delta b^{a}}_{i}\right) \end{array}\right],$$

where $\Lambda_{i,j}^{s}$ denotes the information matrix, and we use the sub-block of $\Lambda_{i,j}^{\mu }$.

For the relative pose between two keyframes *i* and *j* given as $p_{i,j}=R\left(q_{i}\right)\left({}^{w}p_{j}-{}^{w}p_{i}\right)$ and $q_{i,j}={}_{d}^{w}q_{i}^{*}{}_{d}^{w}q_{j}$, the relative pose costs in PGO are given as follows:

(30) $$C_{i,j}^{\mathrm{rel}}=e^{\mathrm{rel}}{\left(i,j\right)}^{⊤}\Lambda_{i,j}^{\mathrm{rel}}e^{\mathrm{rel}}\left(i,j\right)$$

(31) $$e^{\mathrm{rel}}\left(i,j\right)=\left[\begin{array}{c} p_{i,j}-{\hat{p}}_{i,j}\\ 2\;*\mathrm{Vec}(q_{i,j}\;{\hat{q}}_{i,j}^{*}) \end{array}\right]$$

where (${\hat{p}}_{i,j},{\hat{q}}_{i,j}$) is the relative pose constraint between keyframe *i* and *j* in the current map, Vec(q) returns the vector (imaginary) part of q, and $\Lambda_{i,j}^{\mathrm{rel}}$ is the information matrix from the keyframe pose covariance. We define the optimization cost for a new state $S_{pgo}$ by combining Equations (28) and (31) for whole keyframes *n* as follows:

(32) $$S_{pgo}^{*}=\underset{S_{pgo}}{\mathrm{argmin}}\left\{\sum_{i,j\in k}{C^{\mathrm{rel}}}_{i,j}+\sum_{k}C_{k,k+1}^{s}\right\},\;\;\;k\in \left[0,n\right].$$

### 5.3. Convergence Check

**Figure 9:**
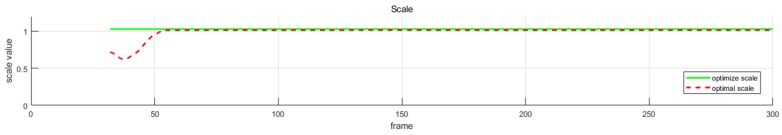
The optimized scale variable for the sequence MH (Machine Hall) 01. The optimal scale value is computed by aligning the estimated visual trajectory with the ground-truth poses via Sim(3) [42]. Our bootstrapping algorithm estimates reliable initial scales within the 50th frame, then updates the local scale by Equation (26) incrementally. It can be verified that the estimated scales are very close to the optimal values.

While the proposed scale and gravity optimization can be calculated in real time at the moment of insertion of a new keyframe, we need to determine when to update the current map with the optimized parameters to initialize the VIO process. We use two ways to measure the convergence: the covariance of $S_{pgo}^{*}$ and the variance of the global scale variable. $S_{pgo}^{*}$ is the optimal solution for the states $S_{pgo}$ for the maximum likelihood estimation. Then, the covariance of $S_{pgo}^{*}$ is given by,

(33) $$C\left(S_{pgo}^{*}\right)={\left(J{\left(S_{pgo}^{*}\right)}^{⊤}\;J\left(S_{pgo}^{*}\right)\right)}^{-1}$$

where $J(S_{pgo}^{*})$ is the Jacobian of Equation (32) at $S_{pgo}^{*}$. One way to measure the quality of the solution for the non-linear least squares problem is to analyze the covariance of the solution. For a non-linear cost function of the state S and the maximum likelihood estimate $S^{*}$, $J(S_{pgo}^{*})$ can be computed as the Jacobian of Equation (32) at the optimal state $S_{pgo}^{*}$. We apply the optimized scale and gravity to the system initialization when the largest eigenvalue of the optimized covariance $\lambda_{max}\left(C\left(S_{pgo}^{*}\right)\right)$ is less than the threshold $\tau^{cov}$ and the scale variance is less than the threshold $\tau^{var}$ at the same time. Figure 9 shows one example of global scale estimation in the bootstrapping process. In the experiments, the scale and gravity initialization in the bootstrapping stage are estimated to reliable values within 5 s on average for the EuRoC dataset.

### 5.4. IMU Biases Update

After the optimized scale and gravity are applied to the poses, we can calculate the initial IMU biases while fixing all pose variables ${}_{d}^{w}\theta_{i}$ in the optimization using Equation (25). As the biases are updated, pre-integration for the local window keyframes is re-computed. At this point, the bootstrapping of the VIO is complete, and afterwards, the online optimization is performed using the framework presented in Section 4.3. Algorithm 1 shows the overall procedure of our method. Our proposed system runs from the bootstrapping to online visual inertial optimization efficiently. In Section 6, we discuss our results with the other comparison methods and how the estimated metric scale converges to the true values by the proposed bootstrapping.

**Algorithm 1:** Proposed online VIO algorithm.
**Data**: Images, accelerations and gyro**Result**: 6DOF poses and landmarksInitialization: Select 2 keyframes for visual motion-based initialization, and perform visual odometry to estimate relative keyframe motion [43]. Then, calculate the metric scale and gravity by PGO with the IMU factor. Check the convergence of the optimized parameters, and re-propagate the pre-integration factor using the initial bias, scale, and gravity;

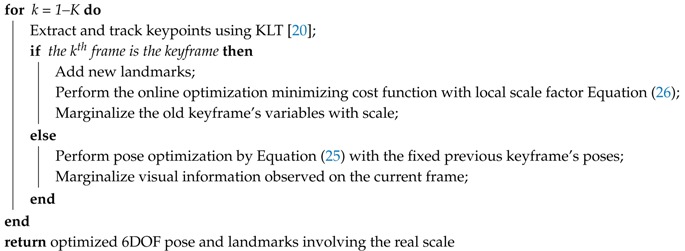




## 6. Experiments

We use the EuRoC [10] dataset, which contains various challenging motions, to evaluate the performance of the proposed algorithm quantitatively. The dataset is collected from the Firefly micro-aerial vehicle equipped with a stereo camera and an IMU at high flying speeds. We use only the left images with inertial sensor data. The sensor data in the EuRoC dataset are captured by a global shutter WVGA monochrome camera at 20 fps and the IMU at 200 Hz. This dataset consists of five “Machine Hall” sequences and six “Vicon Room” sequences, whose difficulties are labeled as easy, normal, and difficult, depending on the motion speed and environmental illumination changes. Both datasets contain the ground-truth positions measured by the Leica MS50 laser tracker and the Vicon motion capture systems, which are well calibrated to be used as the benchmark datasets in various VO/VIO/SLAM applications. The proposed system is implemented in C++ without GPU acceleration and is executed on a laptop with Intel Core i7 3.0 G CPU and 16 GB RAM in real time.

### 6.1. Comparison with the State-of-the-Art Algorithms

We compare the proposed algorithm with the recent state-of-the-art approaches using the same evaluation method by Delmerico and Scaramuzza [33], where the evaluation results of the VIO systems were presented. All parameter settings are kept unchanged in all tests, and the metric is the RMSE position error over the alignment trajectory to the ground-truth pose via SE(3) [44]. Note that, because our proposed method is not a SLAM system, we only compare ours with the systems that do not have loop closing. We directly compare the RMSE results with those of Open Keyframe-based Visual-Inertial SLAM (OKVIS) [5], Robust Visual Inertial Odometry (ROVIO) [45], Monocular Visual-Inertial Systems (VINS-Mono) [17], Semi-direct Visual Odometry (SVO) + Multi Sensor Fusion (MSF) [46,47], and SVO + Georgia Tech Smoothing and Mapping (GTSAM) [29] presented in [33].

OKVIS is an open source VIO system that minimizes the visual re-projection errors for landmarks and IMU measurement with non-linear optimization. It uses a direct integration model without using the IMU pre-integration method. ROVIO is an EKF-based VIO system that updates the pose state using multi-level patches around feature points with propagated IMU motion and minimization of photometric errors. VINS-Mono is similar to OKVIS as it uses the non-linear optimization based on a sliding window, but it incorporates the IMU pre-integration for relative pose constraints between the keyframes. In addition, the authors propose a loop closure using 4DOF pose graph optimization, which is not included in our comparison. SVO + MSF is an algorithm that combines semi-direct visual odometry (SVO) [47], which can quickly estimate the frame poses based on visual patches and IMU measurement, with the EKF framework. Note that it needs manual initialization using extra sensors. SVO + GTSM optimizes structureless visual reprojection error with IMU pre-integration, performing full-smoothing factor graph optimization by [14]. These methods differ from usage of visual terms (re-projection and photometric error), IMU terms (IMU pre-integration and direct integration), and minimization methods. Unlike with SLAM systems, VIO does not use re-localization and loop closing. Momentary failures in pose estimation, e.g., due to fast motion or dramatic illumination changes in Vicon Room1-03 (V1-03) or V2-03, can result in large pose errors in a long trajectory, and this is useful in evaluating the robustness of the systems.

**Figure 10:**
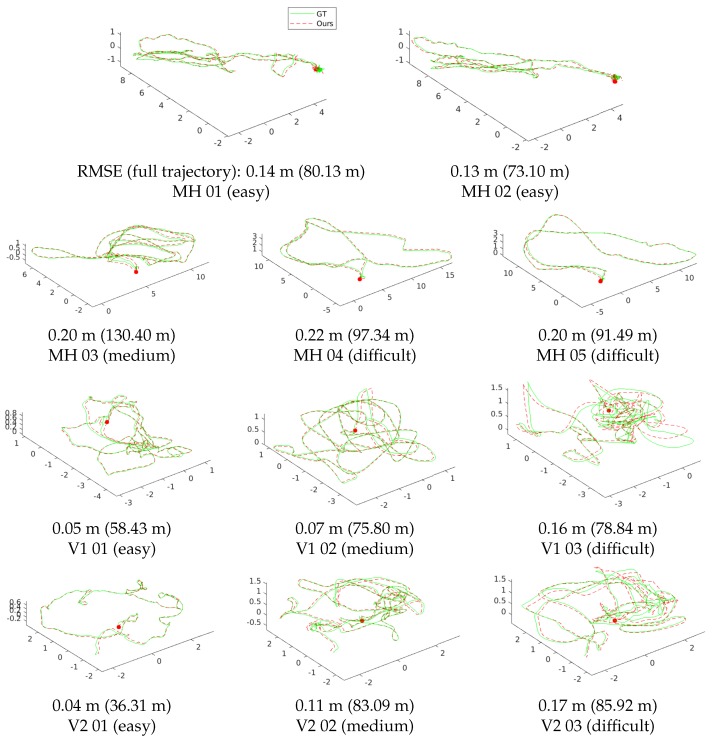
Comparison trajectory result of the proposed method with the ground-truth. Estimated trajectories are aligned to the ground-truth pose via SE(3). The green line represents the ground-truth trajectory, and the red dashed line is ours. For overall sequences, the proposed method estimates the accurate poses without any failure cases in the tightly-coupled optimization framework with a robust initialization method using relative pose constraints.

Table 1 shows the RMSE of the proposed algorithm and the state-of-the-art VIO systems in terms of the estimated full trajectories of EuRoC. Figure 10 shows the estimated trajectories of our method and the ground-truth poses. Our system works robustly and accurately in all sequences without any failures. ROVIO, VINS, and OKVIS operate robustly in all sequences, but show low accuracy at V2-03, which is difficult to initialize robustly due to fast motion, and MH-05, which contains a night-time outdoor scene. SVO + GTSAM achieves superior performance in the “Machine Hall” sequences with far features with illumination changes; however, it fails to estimate correct trajectories of some “Vicon Room” sequences with fast motion (V1-03, V2-02∼03). Our algorithm performs well in MH-04∼05 and V1∼2-03, which are the most difficult sequences with dramatic illumination changes, motion blur, and dark illumination. Accurate scale and gravity initialization helps with the reliable estimation of the bias, and it in turn enables estimating exact poses even when the feature tracking is unstable. We have the best performance for overall without any failure cases, due to our tightly-coupled optimization framework with the robust initialization method using relative pose constraints. The most important aspect of the UAV applications is to estimate the vehicle ego-motion stably for the entire running. The proposed method is suited for this purpose since it can yield accurate poses from the global metric scale and gravity estimation using visual and inertial information together.

### 6.2. Bootstrapping Experiments

**Figure 11:**
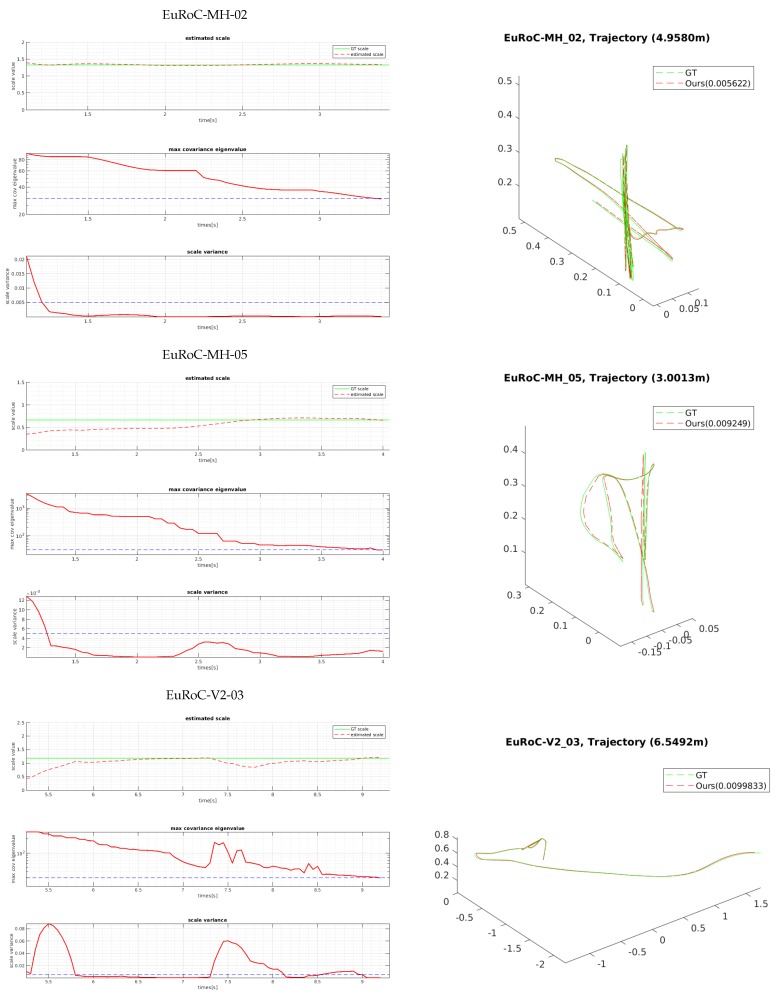
The scales and the two variance-based confidence metrics at the bootstrapping stage, as well as the estimated and ground-truth trajectories of three EuRoC sequences are shown. The left graphs show the estimated scale, the maximum eigenvalues of the covariance, and the variance of the scale parameter from top to bottom. When the bootstrapping starts, the uncertainty of the pose and scale is large, and it is reflected in the metrics. As more visual and inertial observations become available, the variances decrease, and the bootstrapping ends when both go below the thresholds (shown in blue dashed lines). The right 3D plots are the ground-truth (green) and estimated (red) trajectories of the initial 15-s period. Our adaptive bootstrapping successfully finishes within 5 s for the challenging EuRoC sequences, and the RMSE pose errors after 15 s are less than 0.01 m.

**Figure 12:**
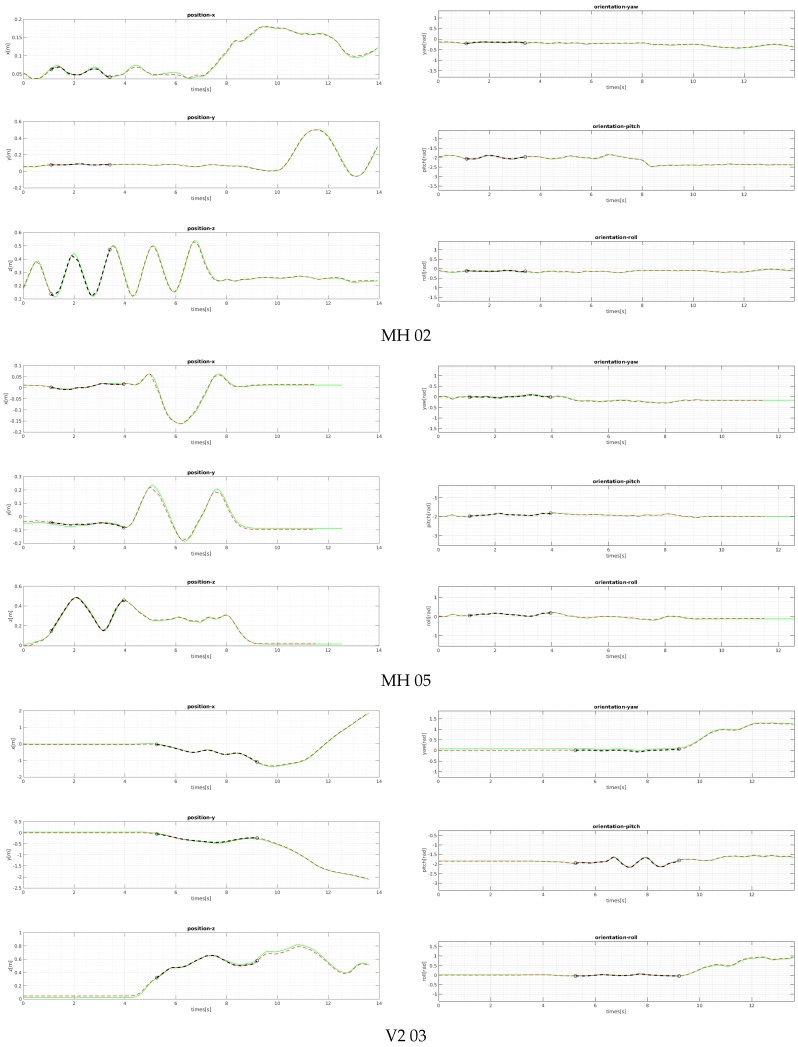
The comparison of the ground-truth positions and orientations with ours. The ground-truth values are plotted in green, and the estimated values are the red dashed lines. The black lines represent the section where the bootstrapping takes place. Note that it starts when there exists enough motion and finishes when the two confidence metrics are satisfied (Figure 11). The graphs show that the our adaptive proposed initialization method estimates the scale and bias parameters reliably and accurately.

**Figure 13:**
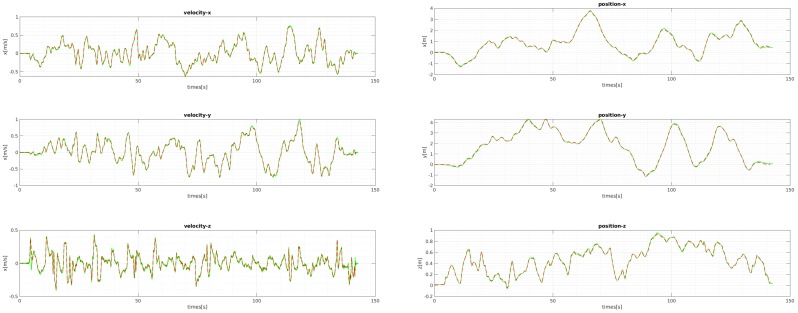
The comparison of the ground-truth velocities and positions with ours for V1 01. The ground-truth values are plotted in green lines, and the estimated values are the red dashed lines. The positions and velocities computed by the estimated scale from the bootstrapping and updated by the local scale parameter align well with the ground-truth.

We evaluated the proposed bootstrapping using a few challenging datasets of MH 02 (easy), MH 05 (difficult), and V2 03 (difficult). Figure 11 and Figure 12 show the plots of the bootstrapping progress, as well as the trajectory and individual parameters of our estimation vs. the ground-truth for the first 15 s. With insufficient short-term initial poses, the estimated scale is very likely to be incorrect and unstable, but it is also not desirable for the bootstrapping to take too long. The proposed two variance-based metrics can effectively determine if the scale can be estimated reliably, and they can be computed easily from the PGO with IMU pre-integration. Even when the visual observation is noisy and insufficient in MH 05 and V2 03 due to fast motion, our proposed bootstrapping estimates the metric scale within 5 s. Furthermore, the estimated initial scale in short bootstrapping time is gradually refined by the local scale factor throughout in the online visual-inertial optimization framework for improved pose estimation. Figure 13 shows the plots of the comparison for the positions and velocities of V1-01. Our proposed initialization and online scale update method successfully estimates positions and velocities, which involve the metric scale.

The estimated scale graphs of Figure 11 show that our estimated scale variable converged to the optimal scale in bootstrapping. The true scale value is computed by aligning the estimated visual trajectory with the ground-truth poses via similarity transformation [42]. The two graphs below them show the convergence-check parameters in bootstrapping, which are described in Section 5.3. Experimentally, we set the maximum eigenvalue of the covariance $\tau^{cov}$ to 30 and the scale variance $\tau^{var}$ to 0.005. Note that the variances can be estimated when there exists meaningful motion. For example, in Figure 11, the first 0.6 (MH 02)∼5.1 (V2 03) seconds are not used. When both metrics drop below the thresholds, bootstrapping is finished, and the estimated scale is applied to the entire trajectory. In contrast, ref. [6] is designed to wait for 15 s to find the initial variables (scale, gravity, and biases) by the closed-form solution. Once the parameters are found at the beginning, they are not updated afterwards; thus, if the calculated scale variable is not accurate, the following pose estimation can fail completely. Compared to [6], our method provides an adaptive and reliable bootstrapping.

Figure 12 shows our position and orientation estimates after bootstrapping compared with the ground-truth. The proposed method provides reliable scaled position and aligned orientation; thus, it is suited for various robotics systems in the real world.

## 7. Conclusions

In this paper, we propose a robust and accurate monocular visual inertial odometry system, which can be applied to UAVs in unknown environments. Even when the initial motion is not known and constrained, we optimize the relative motion with the IMU pre-integration factors to solve the highly non-linear problem effectively and estimate the reliable states with convergence criteria to bootstrap the system. We also estimate the local scale and update it with marginalization of old keyframes to overcome the limitation of the sliding window approach. We evaluate the robustness and accuracy of the proposed method with the EuRoC benchmark dataset, which contains various challenges, and show that ours outperform the state-of-the-art VIO systems.

The problem of state estimation of UAVs is a challenging research topic due to its dynamic motion and interaction with the unknown environment. Therefore, we are interested in further extending the algorithm with additional sensors for stable operation in the real world. In addition, we are planning the dense map reconstruction from the reliable device poses estimated from various sensors. The high-density reconstruction of the environment can be applied to various applications such as obstacle detection, re-localization, and 3D object tracking, which will help UAVs become more practical.

## Figures and Tables

**Table 1:** Average distance error on the EuRoC dataset (unit: m).

EuRoC Sequence	Ours	SVO + MSF [46]	OKVIS [5]	ROVIO [45]	VINS-Mono [17]	SVO + GTSAM [29]
MH 01 (easy)	0.14	0.14	0.16	0.21	0.27	0.05
MH 02 (easy)	0.13	0.20	0.22	0.25	0.12	0.03
MH 03 (medium)	0.20	0.48	0.24	0.25	0.13	0.12
MH 04 (difficult)	0.22	1.38	0.34	0.49	0.23	0.13
MH 05 (difficult)	0.20	0.51	0.47	0.52	0.35	0.16
V1 01 (easy)	0.05	0.40	0.09	0.10	0.07	0.07
V1 02 (medium)	0.07	0.63	0.20	0.10	0.10	0.11
V1 03 (difficult)	0.16	X	0.24	0.14	0.13	X
V2 01 (easy)	0.04	0.20	0.13	0.12	0.08	0.07
V2 02 (medium)	0.11	0.37	0.16	0.14	0.08	X
V2 03 (difficult)	0.17	X	0.29	0.14	0.21	X
Overall	**0.13**		0.23	0.22	0.16	
